# Supplementary material for: Origami folding: Taxing resources necessary for the acquisition of sequential skills
Source: PLoS One. 2020 Oct 5;15(10):e0240226. doi: 10.1371/journal.pone.0240226 (PMC7535859; doi:10.1371/journal.pone.0240226)
Supplement: S1 Appendix — (DOCX) [file pone.0240226.s001.docx]

**Origami folding: Taxing resources necessary for the acquisition of sequential skills**

**S1 Appendix**

**S1 Table. Average reaction time (sec) and error rates (percentage) of each figure across all participants.**

| Trial | Step 1 | Step 2 | Step 3 | Step 4 | Step 5 | Step 6 | Step 7 | Step 8 | Step 9 |
| --- | --- | --- | --- | --- | --- | --- | --- | --- | --- |
| **Mean RTs in sec Mean (SD)** | | | | | | | | | |
| **Chair** | | | | | | | | | |
| 1 | 21.97 | 24.76 | 34.61 | 32.33 | 13.14 | 38.02 | 29.66 | 12.06 |  |
|  | (10.44) | (12.42) | (19.89) | (20.65) | (15.83) | (25.34) | (18.70) | (11.37) |  |
| 2 | 19.42 | 21.25 | 27.57 | 26.58 | 9.44 | 28.91 | 18.72 | 8.59 |  |
|  | (10.87) | (11.12) | (16.09) | (17.06) | (14.34) | (19.13) | (10.97) | (7.27) |  |
| 3 | 15.49 | 20.65 | 23.07 | 21.90 | 5.37 | 29.08 | 15.91 | 6.09 |  |
|  | (8.16) | (12.59) | (13.92) | (14.21) | (6.83) | (18.69) | (10.05) | (4.91) |  |
| 4 | 16.59 | 19.55 | 21.00 | 20.59 | 4.24 | 22.58 | 14.56 | 6.00 |  |
|  | (12.15) | (10.99) | (12.59) | (13.85) | (3.54) | (14.17) | (11.86) | (5.19) |  |
| **Box** | | | | | | | | | |
| 1 | 19.07 | 23.14 | 28.64 | 18.16 | 23.99 | 56.92 | 30.93 | 38.83 | 11.66 |
|  | (8.79) | (11.40) | (14.33) | (18.30) | (15.13) | (36.69) | (27.89) | (24.93) | (11.88) |
| 2 | 16.74 | 21.72 | 21.59 | 11.22 | 23.60 | 33.64 | 17.76 | 26.78 | 8.31 |
|  | (7.28) | (10.52) | (10.66) | (10.04) | (17.97) | (23.97) | (17.21) | (21.54) | (10.12) |
| 3 | 15.14 | 20.03 | 17.82 | 9.29 | 16.61 | 29.60 | 12.41 | 22.55 | 7.22 |
|  | (6.86) | (9.25) | (10.36) | (10.17) | (9.92) | (21.93) | (13.56) | (17.91) | (7.46) |
| 4 | 15.99 | 19.16 | 18.10 | 7.14 | 14.99 | 26.36 | 10.93 | 15.94 | 6.80 |
|  | (8.00) | (9.10) | (11.51) | (5.71) | (8.93) | (19.74) | (11.74) | (12.74) | (7.26) |
| **Penguin** | | | | | | | | | |
| 1 | 28.76 | 6.78 | 44.69 | 34.75 | 39.05 | 57.21 | 16.94 | 20.53 | 19.47 |
|  | (12.97) | (4.30) | (21.04) | (17.41) | (19.00) | (32.58) | (15.12) | (15.36) | (13.91) |
| 2 | 22.26 | 4.74 | 34.20 | 23.54 | 33.93 | 44.34 | 12.56 | 11.32 | 11.99 |
|  | (12.94) | (2.71) | (18.74) | (11.47) | (21.04) | (28.88) | (10.77) | (7.02) | (10.63) |
| 3 | 19.55 | 3.75 | 31.05 | 22.75 | 29.25 | 37.94 | 9.50 | 9.74 | 9.71 |
|  | (11.9) | (2.51) | (19.54) | (16.46) | (19.79) | (25.94) | (5.66) | (11.15) | (8.36) |
| 4 | 16.96 | 2.68 | 26.15 | 19.17 | 23.77 | 35.15 | 8.10 | 6.98 | 8.06 |
|  | (10.48) | (1.8) | (17.41) | (13.18) | (14.86) | (25.26) | (5.96) | (4.59) | (7.21) |
| **Butterfly** | | | | | | | | | |
| 1 | 27.00 | 17.96 | 30.67 | 29.55 | 9.39 | 17.79 | 34.71 | 28.85 | 36.55 |
|  | (10.35) | (7.8) | (21.02) | (21.22) | (15.62) | (9.78) | (22.32) | (25.05) | (26.51) |
| 2 | 21.57 | 16.72 | 23.72 | 23.26 | 6.25 | 14.38 | 29.05 | 21.73 | 26.77 |
|  | (12.36) | (12.54) | (21.62) | (16.69) | (6.35) | (9.43) | (20.74) | (17.9) | (17.04) |
| 3 | 18.93 | 14.32 | 21.03 | 18.15 | 3.69 | 12.31 | 24.57 | 17.25 | 22.76 |
|  | (9.8) | (11.7) | (19.91) | (14.01) | (2.36) | (9.91) | (18.48) | (18.49) | (17.35) |
| 4 | 20.73 | 12.38 | 16.64 | 16.43 | 5.50 | 9.66 | 18.91 | 9.98 | 17.17 |
|  | (12.68) | (7.89) | (15.55) | (10.6) | (7.17) | (7.39) | (15.47) | (8.05) | (12.1) |
| **Frog** | | | | | | | | | |
| 1 | 24.75 | 16.00 | 48.64 | 26.87 | 5.76 | 22.48 | 22.25 | 16.73 | 33.56 |
|  | (10.87) | (7.85) | (26.61) | (15.68) | (6.36) | (14.29) | (10.93) | (9.06) | (19.98) |
| 2 | 20.35 | 13.51 | 32.98 | 21.33 | 4.90 | 16.27 | 16.76 | 16.47 | 24.05 |
|  | (8.66) | (8.95) | (22.71) | (15.1) | (3.86) | (8.87) | (10.29) | (14.11) | (15.34) |
| 3 | 20.13 | 12.14 | 31.64 | 18.74 | 4.09 | 14.22 | 13.17 | 12.87 | 19.16 |
|  | (9.84) | (8.24) | (22.93) | (13.15) | (5.31) | (9.89) | (6.88) | (9.36) | (13.1) |
| 4 | 19.31 | 11.29 | 27.80 | 14.90 | 4.02 | 12.97 | 13.91 | 11.99 | 16.84 |
|  | (10.16) | (7.55) | (20.32) | (8.82) | (4.65) | (9.11) | (11.19) | (10.19) | (11.77) |
| **Mean of Error Rates in Percentage M (SD)** | | | | | | | | | |
| **Chair** | | | | | | | | | |
| 1 | 0.00 | 1.89 | 16.98 | 24.53 | 16.98 | 49.06 | 30.19 | 20.75 |  |
|  | (0) | (13.74) | (37.91) | (43.44) | (37.91) | (50.47) | (46.35) | (40.94) |  |
| 2 | 0.00 | 0.00 | 5.66 | 11.32 | 3.77 | 20.75 | 7.55 | 3.77 |  |
|  | (0) | (0) | (23.33) | (31.99) | (19.24) | (40.94) | (26.67) | (19.24) |  |
| 3 | 1.89 | 3.77 | 3.77 | 11.32 | 1.89 | 5.66 | 5.66 | 1.89 |  |
|  | (13.74) | (19.24) | (19.24) | (31.99) | (13.74) | (23.33) | (23.33) | (13.74) |  |
| 4 | 0.00 | 1.89 | 5.66 | 9.43 | 1.89 | 5.66 | 5.66 | 0.00 |  |
|  | (0) | (13.74) | (23.33) | (29.51) | (13.74) | (23.33) | (23.33) | (0) |  |
| **Box** | | | | | | | | | |
| 1 | 0.00 | 0.00 | 9.43 | 24.53 | 15.09 | 58.49 | 28.30 | 30.19 | 18.87 |
|  | (0) | (0) | (29.51) | (43.44) | (36.14) | (49.75) | (45.48) | (46.35) | (39.5) |
| 2 | 0.00 | 0.00 | 7.55 | 22.64 | 13.21 | 45.28 | 9.43 | 22.64 | 7.55 |
|  | (0) | (0) | (26.67) | (42.25) | (34.18) | (50.25) | (29.51) | (42.25) | (26.67) |
| 3 | 0.00 | 0.00 | 7.55 | 13.21 | 5.66 | 18.87 | 7.55 | 7.55 | 1.89 |
|  | (0) | (0) | (26.67) | (34.18) | (23.33) | (39.5) | (26.67) | (26.67) | (13.74) |
| 4 | 0.00 | 0.00 | 5.66 | 5.66 | 3.77 | 13.21 | 3.77 | 5.66 | 1.89 |
|  | (0) | (0) | (23.33) | (23.33) | (19.24) | (34.18) | (19.24) | (23.33) | (13.74) |
| **Penguin** | | | | | | | | | |
| 1 | 5.66 | 5.66 | 18.87 | 18.87 | 32.08 | 73.58 | 22.64 | 24.53 | 15.09 |
|  | (23.33) | (23.33) | (39.5) | (39.5) | (47.12) | (44.51) | (42.25) | (43.44) | (36.14) |
| 2 | 3.77 | 1.89 | 15.09 | 9.43 | 16.98 | 54.72 | 11.32 | 7.55 | 5.66 |
|  | (19.24) | (13.74) | (36.14) | (29.51) | (37.91) | (50.25) | (31.99) | (26.67) | (23.33) |
| 3 | 0.00 | 0.00 | 3.77 | 7.55 | 7.55 | 35.85 | 3.77 | 5.66 | 3.77 |
|  | (0) | (0) | (19.24) | (26.67) | (26.67) | (48.41) | (19.24) | (23.33) | (19.24) |
| 4 | 0.00 | 1.89 | 3.77 | 1.89 | 5.66 | 22.64 | 5.66 | 7.55 | 3.77 |
|  | (0) | (13.74) | (19.24) | (13.74) | (23.33) | (42.25) | (23.33) | (26.67) | (19.24) |
| **Butterfly** | | | | | | | | | |
| 1 | 0.00 | 1.89 | 33.96 | 22.64 | 9.43 | 9.43 | 47.17 | 43.40 | 45.28 |
|  | (0) | (13.74) | (47.81) | (42.25) | (29.51) | (29.51) | (50.4) | (50.04) | (50.25) |
| 2 | 20.75 | 9.43 | 3.77 | 3.77 | 26.42 | 13.21 | 18.87 | 0.00 | 0.00 |
|  | (0) | (0) | (40.94) | (29.51) | (19.24) | (19.24) | (44.51) | (34.18) | (39.5) |
| 3 | 0.00 | 0.00 | 20.75 | 9.43 | 3.77 | 3.77 | 26.42 | 13.21 | 18.87 |
|  | (0) | (0) | (29.51) | (0) | (13.74) | (0) | (39.5) | (23.33) | (37.91) |
| 4 | 0.00 | 0.00 | 3.77 | 1.89 | 0.00 | 1.89 | 11.32 | 5.66 | 13.21 |
|  | (0) | (0) | (19.24) | (13.74) | (0) | (13.74) | (31.99) | (23.33) | (34.18) |
| **Frog** | | | | | | | | | |
| 1 | 1.89 | 0.00 | 28.30 | 16.98 | 3.77 | 18.87 | 22.64 | 20.75 | 32.08 |
|  | (13.74) | (0) | (45.48) | (37.91) | (19.24) | (39.5) | (42.25) | (40.94) | (47.12) |
| 2 | 1.89 | 1.89 | 22.64 | 7.55 | 3.77 | 9.43 | 5.66 | 18.87 | 20.75 |
|  | (13.74) | (13.74) | (42.25) | (26.67) | (19.24) | (29.51) | (23.33) | (39.5) | (40.94) |
| 3 | 0.00 | 0.00 | 11.32 | 11.32 | 1.89 | 7.55 | 5.66 | 16.98 | 15.09 |
|  | (0) | (0) | (31.99) | (31.99) | (13.74) | (26.67) | (23.33) | (37.91) | (36.14) |
| 4 | 1.89 | 0.00 | 3.77 | 9.43 | 1.89 | 7.55 | 7.55 | 3.77 | 11.32 |
|  | (13.74) | (0) | (19.24) | (29.51) | (13.74) | (26.67) | (26.67) | (19.24) | (31.99) |

**S2 Table. RTs and error rates of secondary tasks (T2).**

|  | Trial 1 | Trial 2 | Trial 3 | Trial 4 | Average | Diff_Trial1-4_ |
| --- | --- | --- | --- | --- | --- | --- |
| **RTs in ms Mean (SD)** | | | | |  |  |
| Cog. Verbal | 2789 (1533) | 2020 (831) | 1952 (824) | 1746 (632) | 2127 (1083) | **1043 (1344)** |
| Cog. Visuospatial | 2560 (1136) | 2425 (1242) | 2347 (1043) | 2227 (1147) | 2390 (1143) | 333 (1242) |
| Mot. Iso. Tapping | 530 (165) | 506 (119) | 513 (144) | 530 (152) | 520 (145) | -1 (144) |
| Mot. Noniso. Tapping | 500 (168) | 504 (162) | 495 (163) | 488 (151) | 497 (160) | 12 (121) |
| **Error rates in %** | | | | |  |  |
| Cog. Verbal | 8.9% (14.5%) | 6.9% (14.2%) | 5.9% (12.5%) | 5.9% (12.1%) | 6.9% (13.3%) | -3.0% (10.8%) |
| Cog. Visuospatial | 14.6% (17.8%) | 11.9% (15.8%) | 14.3% (17.1%) | 14.4% (19.4%) | 17.5% (26.4%) | -0.2% (16.9%) |
| **Absolute timing** | | | | |  |  |
| Mot. Iso. Tapping | 65.4% (25.4%) | 58.3% (30.1%) | 62.0% (27.4%) | 62% (29.6%) | 61.6% (25.3%) | -3.9% (21.6%) |
| Mot. Noniso. Tapping | 92.8% (11.9%) | 90.9% (14.3%) | 90.0% (15.8%) | 90.5% (15.4%) | 90.9% (12.2%) | -2.1% (15.2%) |
| **Relative timing** | | | | |  |  |
| Mot. Iso. Tapping | 61.30% (24.5%) | 64.90% (24.9%) | 67.80% (28.6%) | 62.90% (27.7%) | 64.3% (26.4%) | -1.7% (25.8%) |
| Mot. Noniso. Tapping | 54.50% (22.5%) | 54% (25.2%) | 57.50% (24.5%) | 51.10% (22.7%) | 54.3% (23.7%) | 3.4% (25.0%) |

Note. Bold cells represent the significant values.

**S3 Table. Comparisons of successive pairs of trials.**

Differences of RTs (sec) and error rates (percentage) between successive pairs of trials in the Origami folding task (Task 1) and in secondary tasks.

| **Origami folding (T1)** | | | | | |
| --- | --- | --- | --- | --- | --- |
|  | **Within-subject variance of RT (sec)** | | | **t-Test two-tailed** | |
|  | Trial 1-2 | Trial 2-3 | Trial 3-4 | Trial 1-2 vs. Trial 2-3 | Trial 2-3 vs. Trial 3-4 |
| Cog. Verbal | 5.61 | 2.88 | 2.07 | **<.001** | **0.02** |
| Cog. Visuospatial | 4.68 | 3.15 | 1.84 | **0.02** | **0.005** |
| Mot. Iso. Tapping | 4.74 | 3.53 | 2.66 | **0.045** | 0.08 |
| Mot. Noniso. Tapping | 5.44 | 3.00 | 2.41 | **0.005** | 0.25 |
| None | 6.82 | 3.19 | 2.68 | **<.001** | 0.30 |
|  | **Within-subject variance of error rate %** | | | **t-Test two-tailed** | |
|  | Trial 1-2 | Trial 2-3 | Trial 3-4 | Rep2-1 vs. Rep3-2 | Rep3-2 vs. Rep4-3 |
| Cog. Verbal | 8.5% | 2.7% | 0.7% | **0.002** | **0.002** |
| Cog. Visuospatial | 9.7% | 5.6% | 3.0% | **0.02** | 0.10 |
| Mot. Iso. Tapping | 6.3% | 3.6% | 3.8% | **0.02** | 0.81 |
| Mot. Noniso. Tapping | 7.9% | 4.2% | 4.7% | **0.008** | 0.74 |
| None | 10.8% | 3.9% | 2.2% | **0.002** | 0.30 |
| **Secondary tasks (T2)** | | | | | |
|  | **Within-subject variance of RT (sec)** | | | **t-Test two-tailed** | |
|  | Trial 1-2 | Trial 2-3 | Trial 3-4 | Trial 1-2 vs. Trial 2-3 | Trial 2-3 vs. Trial 3-4 |
| Cog. Verbal | 683.69 | 372.29 | 301.97 | **<.001** | 0.08 |
| Cog. Visuospatial | 602.69 | 479.13 | 409.72 | 0.21 | 0.22 |
| Mot. Iso. Tapping | 80.31 | 65.86 | 55.25 | 0.14 | 0.29 |
| Mot. Noniso. Tapping | 58.82 | 39.28 | 47.92 | **0.03** | 0.16 |
|  | **Within-subject variance of error rate %** | | | **t-Test two-tailed** | |
|  | Trial 1-2 | Trial 2-3 | Trial 3-4 | Trial 1-2 vs. Trial 2-3 | Trial 2-3 vs. Trial 3-4 |
| Cog. Verbal | 5.2% | 3.8% | 4.8% | 0.07 | 0.23 |
| Cog. Visuospatial | 7.0% | 6.1% | 8.3% | 0.54 | 0.09 |
| Mot. Iso. Tapping | 11.8% | 10.3% | 13.4% | 0.39 | 0.10 |
| Mot. Noniso. Tapping | 12.6% | 12.9% | 13.0% | 0.87 | 0.97 |

Note. Bold cells represent the significant values.
