# Supplementary material for: Origami folding: Taxing resources necessary for the acquisition of sequential skills
Source: PLoS One. 2020 Oct 5;15(10):e0240226. doi: 10.1371/journal.pone.0240226 (PMC7535859; doi:10.1371/journal.pone.0240226)
Supplement: Attachment — Submitted filename: Reviewers.docx [file pone.0240226.s002.docx]

Manuscript: PONE-D-20-08555
Title: Origami folding: Memory load effects on acquisition of sequential skills

Journal: PLOS One

**Response to Reviewer #1**

*1. Using within-subject design, the first trial, participants were novices, but for the 2nd or 3rd and the 4th trials, whether participants have been proficient for the primary task then more memory resources have left for secondary task? Therefore, the secondary task for the 2nd, the 3rd and the 4th is not under the same situation of the 1st trial.*

Reply: Thank you for pointing this out. We studied acquisition of serial order under memory load and indeed memory load should have less influence the more proficient participants are with a specific Origami figure. We now mention in the Discussion that across trials working memory should be taxed less and less.

*2. Authors may cite some previous paper folding studies dealing with the acquisition of sequential skills, any papers there or this study is the first study?*

Reply: To our knowledge, our study is the first study using paper folding to examine how sequential skills are acquired through practice. We have mentioned it in the Introduction under the section Origami Folding Task as well as in the Discussion.

*3. Any results comparing the folding of box, chair, frog etc.?*

Reply: We have added the results (reaction times and error rates) comparing folding each figure in the Result section and Appendix in the manuscript (see Fig 2 and S1 Table in Appendix).

*4. Whether folding the different shapes will impose the same level of difficulty?*

Reply: Please see the reply to Comment 3. We have added it to the Results section. Different shapes as well as different steps within a figure can impose different levels of difficulty. The different levels of difficulty might influence the effect of memory load. However, we used the design of Latin-square table, so that the different shapes are combined with all kinds of memory load.

*5. Memory load rather than memory loads --- line 49*

Reply: We have corrected it.

6. One folding step rather than a folding step --- line 109

Reply: We have corrected it.

7. Any hypotheses before conducting the experiment? --- Research Question section

Reply: Two hypotheses were added under the Research Question section.

**Response to Reviewer #2**

*1. Thank you for the submission. The design of the study is interesting, however, I am very confused with the aim of the study. The major reason might be that the research question and conclusion are not coherent with the methodology as well as the analysis.

In the research question section, the aim was made explicitly on “using dual-task paradigm to dissociate the memory load on execution from acquisition”.*

Reply: Thank you for pointing this out. We explained the research question now more clearly about and added the hypotheses. We aimed at comparing how different variants of memory load affect performance in Origami folding and change in performance across trials with practice on a specific figure.

*2. One would expect to see comparison between execution and acquisition.*

Reply: We now acknowledge in the Discussion that further studies would use another research strategy to dissociate memory load effects on practice vs. on performance. (A) a group practices with no memory load and is tested with memory load. (B) a group practices with memory load being tested with memory load and. (C) a group practices and is tested without memory load. The drawback of this strategy would be that participants at test phase in part need to accustom to the novel situation of memory load being present, which might be an extra factor to consider.

*3. However, the actual analyses were on interaction effects between secondary task and number of trials. To be exact, the author conducted repeated measure separately on various the secondary tasks comparing the effect between with and without the secondary task on an individual students’ completion time across different tasks (yet what is 4 repetition in here?).*

Reply: Thank you for pointing out the confusing term. Before the experiment, participants received the folding instruction and did not fold. During the experiment, they folded 4 times. We thus corrected the term “repetition” into “trial”. One trial means one run of folding an Origami figure.

*4. Learning time or performance is not reported at all.*

Reply: The learning time and error rates of Origami-folding task (T1) were reported in Table 2. The learning time or performance of each figure are added to S1 Table in Appendix.

*5. The author stated “There was no speed-accuracy tradeoff” but it doesn’t mean that the completion time is equivalent to accuracy. Also, as the author described on page 16, RT is related to the task complexity. Of course there is a main task effect in the result. Later in the conclusion, the author stated “main effect of secondary task type (indicating performance)” which added even more confusion… so what are being tested?*

Reply: Thank you for pointing this out. We tried to further clarify this. The main effect of task is not about the specific Origami figure it is about a kind of memory load being present vs. absent. To avoid the confusion, we have changed “task type” into “load type”.

*6. In addition to that, Line 72-73 & 77-78, added more confusion in understanding what is being tested in here. In general, how well one acquire a skill is measured by performance. However, the first statement sounds like they are two different things and thus they are being compared in this study. Then in the later statement, the author tried to compare performance with change of performance which is invalid comparison. These two statements are confusing.*

Reply: Thank you for this comment. We tried to further clarify how we can assess skill acquisition. How well one acquires a skill can be estimated by change in performance from Trial 1 to Trial 4. It would be interesting to know how close to the asymptote participants were brought by the 4 numbers of trials that we granted the participants. We acknowledge in the Discussion that a study with (much) more than 4 trials per Origami would be useful to estimate asymptotes. In S3 Table in Appendix, there is a comparison between Trial 1 to Trial 4. As there was still an improvement from Trial 3 to Trial 4, three trials are not sufficient to reach the asymptote.

*7. Other than the unclear aim, in the procedure, page 15 line 349 – 358, participants who fold the Origami wrongly were given more time and chance to learn and practice again. This is NOT a fair learning condition. Thus I won’t be surprised when the author claimed Line 462-464 “we did not observe cases where a dual task variant influenced performance while it did not affect learning (i.e. change in 464 performance)”.*

Reply: Indeed, one could argue that we take a conservative approach and potentially underestimate the learning effects (i.e., difference between Trial 1 and 4) as we helped participants to overcome large difficulties encountered early on. We now briefly mention in the Discussion that support after errors was necessary to secure that participants would be able to complete four trials per Origami figure (rather than that some participants would give up within the first trial of a particular figure).

*8. Also, in the analysis (page 17), when the author wrote “ANOVA …2x4…(results) revealed”, I assumed this was a two-way ANOVA and was about to comment on the abnormal degree of freedom. But soon I saw Greenhouse Geisser-correction was applied, then I realised this is actually a repeated-measures ANOVA, and everything makes a lot more sense. It would be a lot clearer if the author can describe clearly right at the beginning. Furthermore, it is recommended to test the assumption of sphericity before the main analysis.*

Reply: Thank you for pointing this out. We now make our strategy of analysis more explicit upfront.

*9. In the result section, I am not surprised that there is no effect for auditory secondary task. The core of dual-task paradigm is to have a secondary task that take the same working memory resources with the primary task. Learning Origami on the screen is basically a visual task which has nothing to do with auditory loop. Moreover, I do see the value of this study if the aim of the study is to identify which secondary test would potentially affect the learning of origami (which is actually closer to the running head). The author conducted the study with different secondary task, and it’s a shame that the effect of all the secondary task are not compared directly. Yet, in order to achieve this, it would require the author to rewrite (and maybe even re-analysis) the whole article with a clearer (and actually different) aim.*

Reply: Thank you for the comment. Our aim is to test the effect of memory load on performance and on learning (improvement of performance through practice) of Origami folding. We therefore compared the performance of Origami folding (with memory load vs. without memory load) in 4 trials (Trial 1 to Trial 4 through practice). In order to make the aim clearer, we explained the research aim more explicitly in the manuscript. We added an overall repeated-measures ANOVA with 4 kinds of memory load (without the condition with no memory load) and 4 trials (time course of practice from Trial 1 to 4) at the beginning of the analyses. The significant main effect suggested that there were strong differences among the memory load variants. Given that we use a collection of different WM tasks, we deem an approach combining the paired analysis (no load vs. one specific load) as most appropriate.

Although learning Origami folding is a visual task, it can still interfere with auditory loop. Previous studies on implicit sequence learning (Roettger et al., 2019; Schumacher & Schwarb, 2009) have shown that an auditory (auditory-verbal) task can significantly interfere with the acquisition of a sequential skill in a spatial (visual manual – screen locations to key locations) task. In addition, Tenbrink and Taylor (2015) mentioned that while folding participants reformulate the instruction in their own words or thoughts and conduct the movements. The integrative model of text-picture integration (Schnotz, 2014) suggests pictorial information can transform into phonological loops to ease the load in the pictorial channel. However, in the current study, we did not observe such detrimental effects (of verbal memory load) on sequence learning task. It can be due to the low taxing of memory load in the verbal task. We asked participants to recognize the change of one letter in a list of 3 letters. Further studies should be conducted with more challenging verbal secondary tasks.

*10. Line 41 inconsistent referencing style*

Reply: We have corrected it.

*11. Line 57, change “showed a increase” to “showed an increase”*

Reply: We have corrected it. *12. Line 57, replace “with” with “when”*

Reply: We have corrected it. *13. Line 68, “vs.”, do you mean “and?”*

Reply: We have corrected “vs.” into “and”.

*14. Line 71, “4 repetitions” or “4 attempts”? The participants folded the shape 4 times, that is 3 repetitions, right? Or the participants could fold the paper while learning but that original attempt didn’t count? Later in table 1 (page 13-14, line 313), the participants actually have to fold the same shape 5 times. This is quite confusing here. Please illustrate clearly.*

Reply: Thank you for pointing the confusion out. We agree that “repetition” is not the best term, as the participants did not fold while learning. They only read the handouts of the folding instructions with all the folding steps. Then they folded 4 times in the experiment. We changed “repetitions” into “trials” and explicitly explained it in the abstract and the manuscript. Trial 1 refers the first run of folding one figure. Trial 2 refers to the second run of folding the same figure.

*15. Line 93-109 go into methods*

Reply: We have integrated this paragraph into methods.

*16. Section “Origami Folding Task” Describe more about the machine of learning origami or sequential motor skills. See reference below.*

Reply: We have added the mechanism of learning origami or sequential motor skills to the manuscript. We have mentioned the importance of Mirror Neuron System. Thank you for the papers that were recommended.

*17. Line 130, for origami task, it is understandable why visuospatial sketchpad is needed as the person is required to see and read the picture. However it is not unclear why phonological loop is also needed for such a visual task. Please elaborate.*

Reply: Thank you for pointing it out. We have explained now more explicitly, why phonological loop can be involved. According to procedure of Origami folding (Tenbrink & Taylor, 2015), people read and reformulate the folding instructions in their own words at first. In accordance with the Integrative Model of Text-Picture Integration (Schnotz, 2014), pictorial information can be transformed from images in pictorial channel into sounds in verbal channel. When the mental representation of the instruction is constructed, the information can be traced back to verbal and pictorial information. One example would be after seeing the picture of one folding step, some participants used their own words explaining which action they should perform.

*18. Line 209, 206 “ [54,* [*https://osf.io/p3tyf/*](https://osf.io/p3tyf/)*]” The web is not working - page not found.*

Reply: The website can be opened by the authors and their friends as well as the first reviewer. We have positive test results from Germany, UK and China. Besides, we have used the Open Science Framework repository for several other papers and other reviewers could open the link in this repository. We are thus not sure why the web link could not be opened by Reviewer 2. When necessary, we can send the program, the data and the folding steps (the documents in the repository) to Reviewer 2 by email.

*19. Line 220 “part of 4 Bachelor of Science theses”? part of 4-year Bachelor of Science thesis? part of the 4 theses for a Bachelor of Science programme?*

Reply: Thank you for pointing out the confusion. They were part of the 4 theses for a Bachelor of Science program.

*20. Page 15 line 360: RT ---- PLEASE present both the spelled-out and the short form when the abbreviation first appeared. I keep questioning why the authors keep using completion time as reaction time for the whole time until the discussion which is the end of the manuscript!*

Reply: Thank you for pointing it out. We have added the spelled-out and the short form in the abbreviation. *21. Reference:
Castro-Alonso, J. C., Ayres, P., & Paas, F. (2014). Dynamic Visualisations and Motor Skills. In W. Huang (Ed.), Handbook of Human Centric Visualization (pp. 551-580). New York, NY: Springer New York.
Wong, A, Marcus, N, Ayres, P, Smith, L, Cooper, G.A, Paas, G.W.C, & Sweller, J. (2009). Instructional animations can be superior to statics when learning human motor skills. Computers in Human Behavior, 25(2), 339–347. doi:10.1016/j.chb.2008.12.012
Wong, A., Leahy, W., Marcus, N., & Sweller, J. (2012). Cognitive load theory, the transient information effect and e-learning. Learning and Instruction, 22(6), 449-457. doi:10.1016/j.learninstruc.2012.05.004*

 	Reply: Thank you for the references and we have cited them in the manuscript.
