# Supplementary material for: Origami folding: Taxing resources necessary for the acquisition of sequential skills
Source: PLoS One. 2020 Oct 5;15(10):e0240226. doi: 10.1371/journal.pone.0240226 (PMC7535859; doi:10.1371/journal.pone.0240226)
Supplement: Attachment — Submitted filename: Response to reviewers.docx [file pone.0240226.s003.docx]

Manuscript: PONE-D-20-08555R1
Title: Origami folding: Taxing resources necessary for the acquisition of sequential skills
Journal: PLOS ONE

Date: 16-Sep-2020
**Response to Reviewer #1**

*1. Thank for the revised version. I really appreciate the revisions, however, I still have concern about my first comment on the design issue. The level of learners' expertise may be a confounding factor in the design....

Would authors like to run another experiment with between-subject design? Although authors have put my concern as limitation, but the reported experiment may have design issues given no another experiment.*

Reply: Thank you for pointing out the potential benefits of a study involving experts. As we were to study the (early parts of) acquisition of Origami folding, we tested novices as they (due to lack of prior knowledge) should show the largest learning gains and they should be affected the most by taxing the cognitive resources potentially relevant for acquiring the sequential skill. By testing participants with expertise, the potential to find out which resources are relevant for acquiring the skill (by trying different secondary tasks) might have been more limited. Experts might rely on many (sub)processes that are automatized (i.e., no longer affected by taxing the resources by a secondary task).

Given that we used a within-subjects design and we largely avoided that between subjects differences might confound our results. Nevertheless, combining a study on (early) practice in Origami folding with an expert-novice comparison seems a promising route for a future study. We have added the suggestion in the discussion.

**Response to Reviewer #2**

*1. This version is made clearer that the authors are actually investigating in the type of cognitive resources rather than the cognitive load induced by learning origami. The previous version focused on the cognitive load which is a slightly different concept from resources. It is recommended to also change the keywords (and probably also the title) so not to confuse other new readers.*

Reply: Thank you for pointing this out. “Memory load” can be misleading, as we used variants of memory loads to tax specific resources. We have changed the keyword of “memory load” into “cognitive resources”. The title has been changed from "Origami folding: Memory load effects on acquisition of sequential skills" to "Origami folding: Taxing resources necessary for the acquisition of sequential skills".

*2. Moreover, another question arose – was the study going to test the cognitive resources that is relevant to learning *motor task through visualisation*, or to learning *procedural skills* as the author stated multiple times? If the aim is the first one, then the experimental setting makes sense. The secondary tasks were visuospatial-related that were relevant to the learning tasks (i.e. motor tasks) and the learning means (i.e. visualisations). Moreover, the author stated a multiple time in the article and also in the reply to the first author that the aim is to study the learning of procedural skills. Then in the experimental setting, the secondary task should be procedural- or sequential- related. That means the secondary tasks should be some tasks that might hinder participants from memorising the correct sequence. Otherwise the conclusion would not follow from the design. This is also the reason why I put "I don't know" about the rigour of the statistical analysis, as the analysis method largely follows from the study aim and hypothesis.*

Reply: Thank you for the comment. We took care to make clear that we assume that visual representations are essential in the skill acquisition we are studying. We now avoid framing this as a procedural skill. We used a secondary task interfering with the visuospatial sketchpad. Given that we study the early part of skill acquisition (rather than automation of motor patterns), it seems reasonable that task performance and learning are mainly guided by visual representations. In line with the theory by Willingham (1998) on the connection of learning and motor control, participants might mainly rely on (and learn based on) visual representations early in practice (i.e. within the scope of our experiment). In work on sequence learning, it has been shown that participants (at least early in practice) learn about which target in space to affect in which order (sequence of positions rather than sequence of finger movements; cf. Willingham et al. 2000). When practice extends across many sessions, finger movements might become more relevant. According to this reasoning, a visual spatial secondary task should be especially potent to interfere with encoding the sequence or retrieving encoded sequence information.

*4. Also, authors replied to the first reviewer that this is the first study – maybe it’s true when looking at learning origami under repetitive practices; but it’s definitely not the first study dealing with acquisition of sequential skill through looking at origami (see the reference that I recommended before).*

Reply: Thank you very much for the comment. We have edited this and include these studies.

*5. In this revised version, there are still some issues with sentence structures. For example, line 52 “when participants concurrently solve[ing] a cognitive memory load task” What is a cognitive memory load task? Cognitive memory load is not an adjective. What type of tasks that you are referring to? Another example in line 55 “Yet an impact of load on performance does not necessarily imply an impact on learning (i.e., improvement of performance across trials with practice)” i.e. (id est) means “in other words, such as”. What does it mean by no impact on learning such as improvement of performance? Or as simple as missing a subject after adjustive in line 75 “As folding Origami can require cognitive as well as motor control,”…etc. With no intention to list out all the language issue, it is recommended to undergo another thorough proofread before re-submission.*

Reply: Thank you very much for the comment. The listed issues have been corrected. Moreover, we have checked the whole manuscript for grammatical and other language issues as well as for redundancies.
